# Transcriptome comparison reveals key candidate genes in response to vernalization of Oriental lily

**DOI:** 10.1186/s12864-016-2955-0

**Published:** 2016-08-22

**Authors:** Wenqi Li, Xiaohua Liu, Yingmin Lu

**Affiliations:** College of Landscape Architecture & China National Engineering Research Center for Floriculture, Beijing Forestry University, No.35 Qinghua East Road, Haidian District, Beijing, 100083 China

**Keywords:** Vernalization, Lily, Transcriptome, RNA-Seq, Flower differentiation

## Abstract

**Background:**

Oriental hybrid lily ‘Sorbonne’, a very important cut flower for lily, is enjoyed great popularity in the world, but it must experience a period of low winter temperature to initiate or accelerate the flowering process. To gain a better understanding of the temperature signaling pathway and the molecular metabolic reactions involved in the vernalization response, a genome-wide transcriptional analysis using RNA-Seq was performed.

**Results:**

188,447,956 sequencing reads was assembled into 66,327 unigenes and showed similarity to known proteins in the Swiss-Prot protein database, and 2,893, 30,406 and 60,737 unigenes aligned to existing sequences in the KEGG, COG, and GO databases. Based on qRT-PCR results, we studied the expression of three signal regulation pathways genes–the plant hormones signal transduction (*LoAP2*, *LoIAA1*, *LoARF10*), the DNA methylation (*LoCMT*, *LoFLD*), and vernalizatin pathway (*LoFLC*, *LoVRN1*, *LoVRN2*, *LoFT*, *LoSOC1*, *LoLFY*, *LoSVP*) in the immature flower buds of Oriental hybrid lily. In addition, we identified two vernalizaiton–related genes (*LoSVP* and *LoVRN1*) from the cDNA library, which appear to be promising candidates for playing key roles in the development and response of flowering in Oriental lily plants, and *LoSVP* had a function in delaying flowering but *LoVRN1*could promote flowering early.

**Conclusions:**

We collected a sample for transcriptome sequencing and comparison when the bulb’s apical meristem was in the time of floral transition when the apical meristem had not converted into the morphological differentiation process, which helped to obtain more genes playing key roles in the floral induction pathways. The upstream and downstream relationship between different genes were forecasted by the analysis of genes’ expression levels in a wide range of time. Future research that is targeted towards how genes interact on each other, which will promote establishing and perfecting the molecular mechanisms of floral induction pathway by vernalization.

## Background

Vernalization is a complicated process of plant development that is essential for plants to grow in unfavorable environmental conditions, which occurs during cold environment, and flowering only occurs some weeks or even some months later when some other conditions, including the presence of certain photoperiods and ambient temperatures, are met [1]. It is a temporary suspension of vernalization for plants of obvious growth containing meristems. The transitions of vernalization are regulated by chilling temperatures and/or short photoperiods. We have learnt some knowledge about the molecular mechanisms indicating vernalization from the model plant *Arabidopsis thaliana* and cereals.

In *Arabidopsis thaliana*, the progressive repression and stable silencing of *FLOWERING LOCUS C* (*FLC*), is central to the vernalization mechanism. *FLC* encoded a MADS domain protein acting as a repressor of flowering [2, 3]. There were also some other genes been found to related to vernalization-responsiveness in *Arabidopsis* [4]. *FLOWERING LOCUS T* (*FT*) and *SUPPRESSOR OF OVER*-*EXPRESSION OF CONSTANS 1* (*SOC1*), two floral promoters, were repressed transcription by *FLC* [5]. That is, *FLC* is highly expressed in plants that have not experienced vernalization [6, 7], and then *FLC* protein was binded to the promoter of *SOC1* and sequences in an *FT* intron to repress transcription of both these genes [6, 7], which delays flowering. In addition, some other target genes or *FLC* orthologues were key to the developmental release that enables flowering [8, 9] in other plant species.

In cereals, there were two main regulatory pathways leading to the transition to reproductive development at the molecular levels–the vernalization pathway and the photoperiod pathway [10–12]. One of the major genes controlling vernalization-induced flowering is *VERNALIZATION 1* (*VRN1*). *VRN1* is similar to *Arabidopsis thaliana APETALA1* (*AP1*), *CAULIFLOWER* (*CAL*) and *FRUITFULL* (*FUL*), which are MADS-box transcription factors and identity genes in meristem [13]. *VRN1* was a major determinant of winter/spring growth habit in cereals. Neither the mechanism behind repression of *VRN1* prior to vernalization in vernalization-requiring plants nor the mechanism by which *VRN1* leads to transition to reproductive development was fully understood. Another major gene controlling vernalization-induced flowering in cereals is *VERNALIZATION 2* (*VRN2*), which is a floral repressor that delays flowering until plants are vernalized [14].

Besides, there was another group of MADS-box genes that have a putative function in the transition to flowering belong to the *SHORT VEGETATIVE PHASE* (*SVP*)-like MADS-box genes. *SVP*-like genes in *A. thaliana*, *Hordeum vulgare*, and *T. aestivum* act as negative regulators of flowering [15, 16]. In *T. aestivum*, *TaVRT2*, a *SVP*-like gene is down-regulated by vernalization and can bind the CArG-box in the *VRN1* promoter and interacts with *VRN1* and *VRN2* proteins [17].

Lilies, monocotyledonous ornamental plants, are one of the most important flowering crops [18], which flowering relies on a combination of integrating effects of endogenous and external signals [19]. Vernalization in Oriental Lilies, which are perennial plants, generally requires a minimum of eight weeks at low temperatures (4 °C). It was indicated that low temperatures could control vernalization in lily. But there was few studies carried out to date on the transcription factors and vernalization pathway in lily. Here we report the use of the RNA-seq approach to identify venalization-related genes during flower development of Oriental lily.

Recently, based on Illumina sequencing technology, RNA-sequencing (RNA-Seq) technology has become the most powerful and popular tool for plants that lack reference genome information, which is less costly, more efficient, and more accurate and sensitive profiles than other techniques [20]. RNA-Seq has been successfully applied to many species, including *Takifugu rubripes* [21], *Streptococcus mutans* [22], *Streptococcus mutans* [23], *Soybean* [24] and tree peonies [25]. Here, we used RNA-Seq technology to characterize and identify the expression of a large number of genes, especially those expressed differentially during vernalization phase. Our study allows us to investigate the genome-wide interaction dynamics of transcription factors and to identify pathways regulated by vernalization in lily flower development.

## Methods

### Total RNA isolation and plant material

**Fig. 1:**
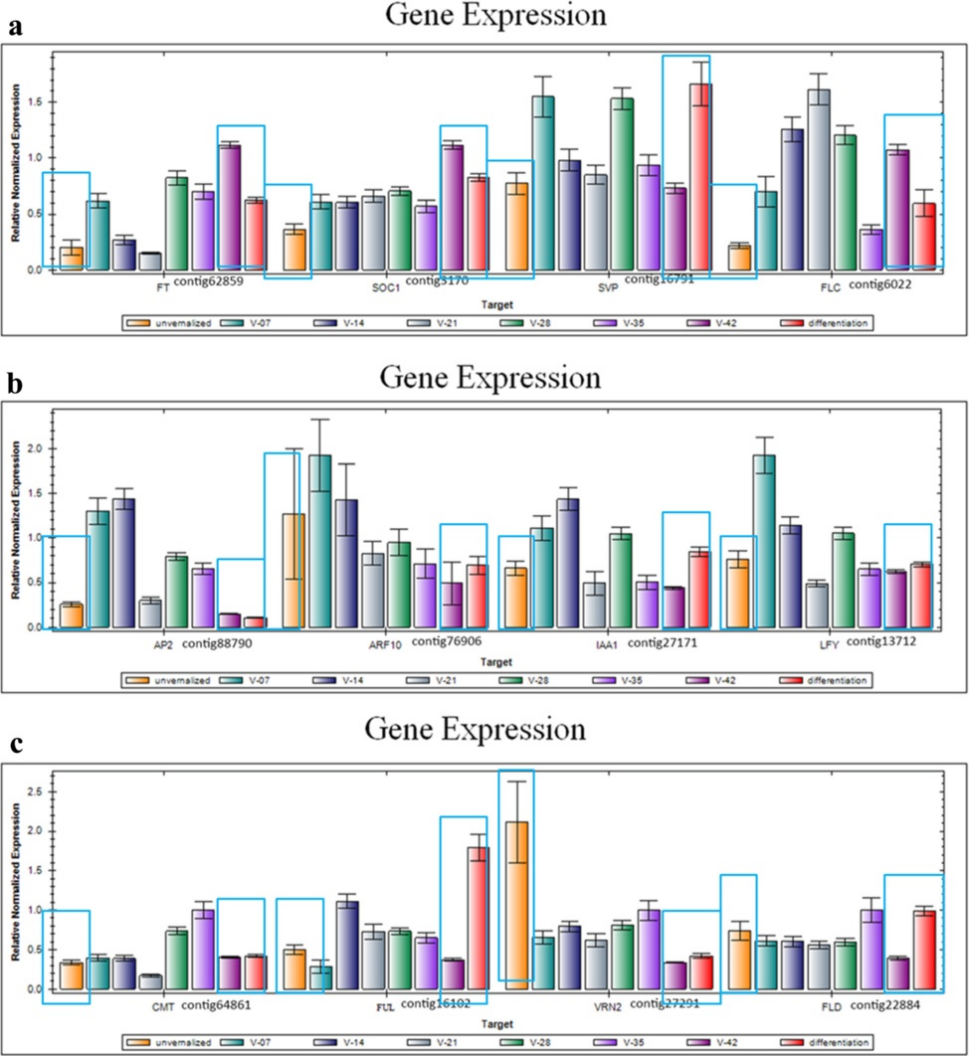
The expression profiles of 12 transcripts in Oriental hybrid lily by the quantitative reverse transcription polymerase chain reaction (qRT-PCR). The Figure indicated the expression of 12 genes from unvernalized stage, different vernalizatin stages (every other week in vernalization) and differentiation stage. The symbol’V-42′ is on behalf of the samples that were obtained after 42-days vernalization, which happens to be the timing of bulbs’dormancy-breaking stage. The expressions of three periods in bule block of each gene by qRT-PCR in order to correspond to the expressions of genes by RNA-seq in Fig. 7

Commercial bulbs of Oriental hybrid lily “Sorbonne” purchased from a local nursery in Yunnan Province after harvest were used. Bulbs were selected with an even size of ca. 20 g. and we stored them in moist sawdust at 4 °C until being planted. The expression of SOC1 gene increases sharply by qRT-PCR after Oriental Hybrid Lily’Sorbonne’ being stored for 42 days which usually indicates the floral transition (Fig. 1). The previous period before the floral transition is the floral induction phase. After the floral transition, the bulb’s apical meristem converts into floral bud morphological differentiation period. At the beginning of the low temperature storage, the unvernalized sample, namely the dormant sample, was collected from the shoot apical meristem (SAM). The vernalized sample, namely the dormancy-breaking sample, was collected from SAM (finished vernalization) after being stored for 42 days, then the flower bud differentiation sample was collected from SAM of squaring stage after being planted. All samples were preserved at-80 °C for RNA isolation. Total RNA was extracted from the tissues with an Easyspin Isolation System of RNAisomate RNA (Aidlab Biotech, Beijing, China). RNA quality was verified by a 2100 Bioanalyzer (Agilent Technologies, Santa Clara, CA, USA). In addition, we used a pooled RNA mixture containing 60 μg RNA from samples to prepare cDNA.

### Library construction and transcriptome sequencing

Each total RNA extract was first treated with RNase-free DNase I (TaKaRa, China) to remove contaminating DNA, and then concentrated the mRNA content by capturing on magnetic oligo (dT) beads. Then second-strand cDNA was synthesized using RNase H, dNTPs, DNA polymerase I, and appropriate buffers with a SuperScript Double-Stranded cDNA Synthesis kit (Invitrogen, Camarillo, CA). Then, cDNA was depurated and settled end repair with an elution buffer and by addition of poly (A). Sequencing adaptors were then ligated to the fragments, and agarose gel electrophoresis used to select the range of fragments suitable for PCR amplification. Sequencing using an Illumina HiSeq™2000 platform was performed at the ShoBiotechnology Corporation (SBC), Shanghai, China following the manufacturer’s protocols. Each sample was sequenced with three cDNA reactions independently as biological replicates.

### Analysis of transcriptome sequencing results

Using scaffolding contig methods with a minimum contig length of ≥400, pre-processing and de novo assembly were carried out including the removal of the adapter sequences, ambiguous inner regions, shorter-than-15-nucleotide sequences, and low quality (Q20, 20) sequences using the SOAP2 aligner [26] configured to allow up two mismatches, discard sequences with “N” s and return all optimal alignments. After assessing the different *K*-mer sizes, the primary unigenes of three samples were assembled, generating the final unigenes which were used for BLASTx searches against Swiss-Prot protein database (2014.05) and the UniProt database (2014.06). Blast2GO program [27] was used to assign GO terms. Finally, 66,327 unigene sequences were aligned into 25 COGs to classify and predict functions. In addition, using BLASTx (E-value threshold 10^−5^), we carried out KEGG annotations based on the KEGG database.

### Identification and bioinformatics annotation of different gene

RPKM method was used to calculate the expression. Using DEGseq, the difference in gene expression between samples were detected [28]. Then according to the Audic & Claverie method [29], a rigorous algorithm was developed to identify genes expressed differentially. FDR was used to decide the threshold of P-value in tests and analysis. When log2 ratio greater than 1 and FDR is less than 0.05 and between the accessions, the genes were regarded as expressed differentially.

### PCR validation by quantitative real-time PCR (qPCR)

Ten candidate genes were selected to determine using quantitative real time PCR (qPCR). The samples were collected from the dormant, different dormancy-breaking stages and different flower bud differentiation stages, as described above. According to the manufacturer’s instructions, with Superscript II reverse transcriptase (Invitrogen, Carlsbad, CA, USA), first-strand cDNA synthesis was performed. qRT-PCR were based on SYBR_Green I (TOYOBO’, Japan) in a DNA Engine Opticon 2 machine (MJ Research, Waltham, MA) and TaKaRa ExTaq RT PCR Kit. A set of gene-specific primer pairs (sequences given in Table 1) was designed by Beacon Designer. The internal reference gene *LoTIP1* (F: CGAAGCCAGAAACGGAGAAGAAT, R: GGGTAGGGTGGATTGGGAAGA) was used as a reference. Each 25 μl qPCR reaction contained 10 ng cDNA, 0.2 μM of each primer and 10 μlSYBR Green PCR master mix, and the amplification regime consisted of an initial denaturation of 95 °C/60 s, followed by 40 cycles of 95 °C/ 15 s, 55 °C/15 s, 72 °C/20 s. All experiments were operated in biological triplicates using the 2^-△△Ct^ method [30], the results were calculated.

**Table 1:** Primers used in real-time quantitative PCR (RT-qPCR)

Unigene id	Protein description	Forward primer sequence (5′–3′)	Reverse primer sequence (5′–3′)	Length (bp)	Correlation between RNA-Seq and qRT-PCR (r^2^)
Contig6022	FLOWERING LOCUS C	CCTGGTGGAGAAGACGATGAGTG	AACAAGATCAGCCGCCAAGTCA	117	0.98
Contig27291	Vernalization 2	GCTGCCAAACCCTGGTCATCA	CATTATGCCTGGTGGTGAGTTCCC	111	0.99
Contig62859	FT-like protein	GCGGCAACGATCTCAGAA	CTCAAATATGGGTTACTGGGACTC	89	0.95
Contig88790	AP2–type transcription factor	GGTTTACTTGGGTGGTTTC	TCCTCCTTCGTCAGATTG	153	0.97
Contig3170	Suppressor of overexpression of CO1	GCCTCGTGAAGAAGA	CTCCAACAGAATCCTC	122	0.94
Contig88645	CBF–like transcription factor	CAACTCGCTGGATGGCTGCT	GCCACTCCGCCACACTCAAT	115	0.98
Contig13712	LFY	CGAAGAAGAACGGGCTGGACTACT	AGGCAGTGGAAGGCGTAGCA	134	0.94
Contig22259	Maf–like protein	CAAGATTCAAACCCGAGATG	CGAGACGATGATGAGGAT	99	0.94
Contig76906	Auxin response factor 10	TAGATGGCACGGGCGAATATACCT	CACACGCAGCCTCTTCACAGT	113	0.97
Contig27171	IAA type protein	GTTCTCTTGCTTCACCATC	ACATACTCCGTTCCATTCA	104	0.98

### Isolation of *LoSVP* and *LoVRN1* genes

The complete sequence of the *LoSVP* and *LoVRN1* were isolated using rapid amplification of cDNA ends (RACE). The CDS sequences in the Oriental hybrid lily EST database were Blast-searched with the homologous fragments of *SVP* in *Arabidopsis thaliana* (AK226537.1) and the homologous fragments of *VRN1* in *Festuca arundinacea* (FJ793194.1) from NCBI. Two genes found in the Oriental hybrid lily EST database have the highest identity with gene (AK226537.1) and gene (FJ793194.1) were named as *LoSVP* and *LoVRN1*, respectively. One microgram of mRNA isolated from SAM was converted into 3′- and 5′-RACE-ready cDNAs with the 3′ and 5′ CDS primers by the SMART RACE cDNA amplification kit (Clontech, Palo Alto, CA, USA).. Specific primers *LoSVP* 5f (5′-CCATGGGACTGAACGATCAGCTTGCG-3′), *LoSVP* 3r (5′-GAATTCCAGTTGCATGTTCTCCTCTG-3′) and *LoVRN1* 5f (5′- CTCTGGATCCCCTCTCATCATCACA-3′), *LoVVRN1* 3r (5′-GAAACACTGGGATCCACTGCCCATG-3′) were designed for amplification of the 5′ and 3′ ends according to the partial sequence of the *LoSVP* and *LoVRN1* of the EST clone, respectively. Reactions were subjected to the following conditions: 94°Cfor 3 min followed by 35 cycles of amplification (94 °C for 30 s, 52 °C for 30 s, 72 °C for 1 min) and a 10-min final extension at 72 °C. Then the PCR products were cloned into pGEMT-T Easy vector. Plasmid DNAs were purified from overnight cultures of three independent clones to sequence for each transformation, and we aligned all resulting sequences with the partial cDNA sequence using the GCG program. Finally, the full-length LoSVP and LoVRN1 cDNA was isolated.

### Analysis of the phylogenetic relationship

Nucleotide sequences were aligned with the program ClustalW [31]. Based on Poisson correction model, neighbor-joining analyses of amino acid sequences included in MEGA4 were used [32] by calculating genetic distance. By bootstrapping, we tested the confidence of reconstructed clades [33]. As a rule, nodes with bootstrap values greater than 70 are significantly supported with 95 % probability [34]. The Genbank accession numbers for amino acid sequences of the *SVP* gene in other plants used are *Theobroma cacao* (XP 007047796), *Populus trichocarpa* (XP 002310310), *Paulownia kawakamii* (AAF22455.1), *Vitis vinifera* (XP 002269295), *Vitis vinifera* (AFC96914.1), *Malus domestica* (ABD66219.1), *Brassica rapa* (ABI96182.1), *Brassica napus* (AFM77910.1), *Capsella rubella* (XP 006294891), *Arabidopsis thaliana* (AFU85632.1), *Arabidopsis thaliana* (BAE98676.1), *Petunia* × *hybrida* (ACV74250.1), *Actinidia chinensis* (AFA37963.1), *Actinidia chinensis* (AFA37967.1), *Elaeis guineensis* (AAW66885.1). The Genbank accession numbers for amino acid sequences of the *VRN1* gene in other plants used are *Festuca arundinacea* (ACN81330.1), *Lolium perenne* (AEV22381.1), *Triticum aestivum* (ABF57926.1), *Dendrocalamus latiflorus* (AAR32119.1), *Oryza saliva* (Baa94342.1), Zea mays (AFW67591.1), Elaeis guineensis (AAQ03221.1), *Alpinia oblongifolia* (ABS83558.1), *Cymbidium ensifolium* (AFQ31623.1), *Vitis vinifera* (AAT07447.1), *Tulipa gesneriana* (BAJ09453.1), *Lilium longiflorum* (ADT78582.1).

### Arabidopsis transformation and transgenic plants analysis

LoVRN1 and LoSVP were excised from the PGEM-T easy vector using *XbaI* and *SmaI* restriction enzymes and inserted in to the binary vector PBI121 (Clontech) under the control of cauliflower mosai cvirus (CaMV) 35S promoter. After confirmation of the sequence, *Agrobacterium tumefaciens* strain GV1301 competent cells were prepared and transformed by electroporation according to Mattanovich et al. [35]. Arabidopsis thaliana ecotype Columbia (Col) plants were transformed using the floral-dip method [36]. The expression of the transgene was confirmed by DNA gel blot analysis, RT-PCR, and immuno-blotting using anti-PBI121 antibody (Clontech, Japan). Wild type Arabidopsis seedlings and 35S: LoVRN1-sPBI121 T1 Arabidopsis transgenic seedlings and 35S: LoSVP-sPBI121 T1 Arabidopsis transgenic seedlings were grown for three days under 0.5× MS with 1 % sucrose media and then were transplanted into the soil, respectively. Observe and compare the flowering time and characteristics of the flower bud differentiation between the transgenic Arabidopsis T1 plants and wild-type Arabidopsis plants. The blade length and plant fresh weight of the transgenic Arabidopsis T1 plants and wild-type Arabidopsis plants were measured in seedling stage, early blooming stage and seed maturing stage, respectively.

## Results

### Illumina sequencing data and de novo assembly

To enrich the number of genes involved in our transcriptome, cDNA samples were extracted from total RNA isolated from SAM for three libraries (dormant, dormancy-breaking and flower bud differentiation samples). Approximately 50 million for the dormant sample and 137 million for the latter two samples (the dormancy-breaking and flower differentiation samples) were obtained. After data cleaning and stringent quality checks, 115,421,520 raw reads containing a total of 17.7 Gb nucleotides were obtained. The average read size, GC percentage and Q20 percentage were 90 bp, 43.00 % and 93.80 %, respectively. 122,464 contigs were assembled with an average length of 624 bp based on the high quality reads. With an average length of 1,015 bp, we further assembled the contigs into 68,036 scaffolds including 31,486 scaffolds larger than 1000 bp. All de novo assembly yielded 66,327 unigenes with an average length of 1,037 bp (Tables 2 and 3). 30,254 (45.6 % of the total) had significant similarity to known proteins in the Swiss-Prot database. The lack of L. oriential genome and EST information meant that 36,073 (54.4 % of the total) unigenes had no Swiss-Prot annotation (Table 3). To demonstrate the quality of sequencing data, ten unigenes were randomly selected and ten pairs of primers were designed for qRT-PCR, and then the products were confirmed by biological Sanger sequencing.

**Table 2:** Summary of sequencing and assembly data

Sample ID	Raw Reads (MB)	Raw Bases (GB)	Q20 Value (%)	Raw Reads	Quality Trimed	Adaptor Trimed	Number Clean Reads	Clean Ratio
unvernalized	50.8	5.1	98.5	50,824,066	49,454,613	48,900,904	47,268,792	93.0
vernalized	56.5	5.6	98.5	56,501,972	55,146,129	54,528,334	52,872,714	93.6
Flower bud differentiation	81.1	8.1	98.6	81,121,918	79,776,697	78,821,040	76,896,022	94.8

**Table 3:** Summary for the Oriental hybrid lily ‘Sorbonne’ transcriptome

Statistics	Counts	Total Length (bp)	N25 (bp)	N50 (bp)	N75 (bp)	Average Length	Longest (bp)	N%	GC%	Annotation Counts	Annotation Ratio%
Contigs	122,464	76,454,985	1,988	948	451	624	16,373	1.3	42.8		
Primary UniGene	68,036	69,046,293	2,341	1,288	674	1,015	19,791	1.1	43.1		
Final UniGene	66,327	68,760,870	2,390	1,333	693	1,037	19,791	1.2	43.0	30,254	45.6

### Functional classification and gene annotation

**Fig. 2:**
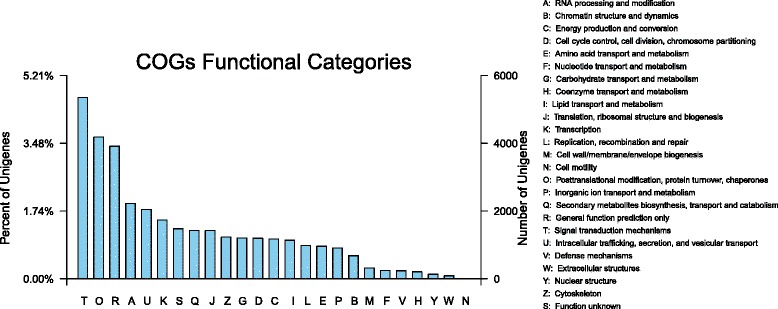
COG classifications in Oriental hybrid lily ‘Sorbonne’

To further evaluate the effectiveness of our annotation process and the completeness of our transcriptome library, the annotated sequences were randomly searched for genes with COG classifications. Of 30,254 hits, 30,406 sequences had a COG classification. Among the 25 COG categories, the cluster for ‘signal transduction mechanisms’ (5351, 17.60 %) represented the largest group, followed by ‘posttranslational modification, protein turnover, chaperones’ (4185, 13.76 %) and ‘general function prediction only’ (3916, 12.88 %). The following categories, ‘nuclear structure’ (137, 0.45 %), ‘extracellular structures’ (92, 0.30 %) and ‘cell motility’ (6, 0.02 %),, represented the smallest groups (Fig. 2).

**Fig. 3:**
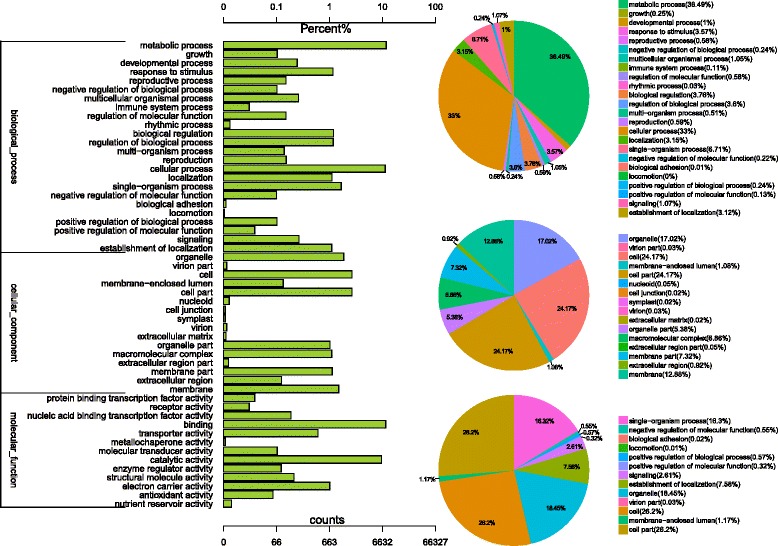
Histogram presentation of Gene Ontology classifications. The results are summarized in three main categories: biological process, cellular component, and molecular function

The GO assignments were also used to classify the functions of the predicted lily genes. Based on sequence homology, 15,425 sequences can be categorized into 53 groups (Fig. 3). In each of the three main GO classifications, the ‘metabolic process’, ‘cell part’ and ‘binding’ terms were dominant, respectively. We also found a high percentage from the ‘cellular process’, ‘membrane-enclosed lumen’ and ‘catalytic activity’ categories, but few from ‘metallochaperone activity’, ‘cell junction’ and ‘locomotion’ (Fig. 3). The GO analysis indicated that the identified genes were associated with various biological processes.

**Fig. 4:**
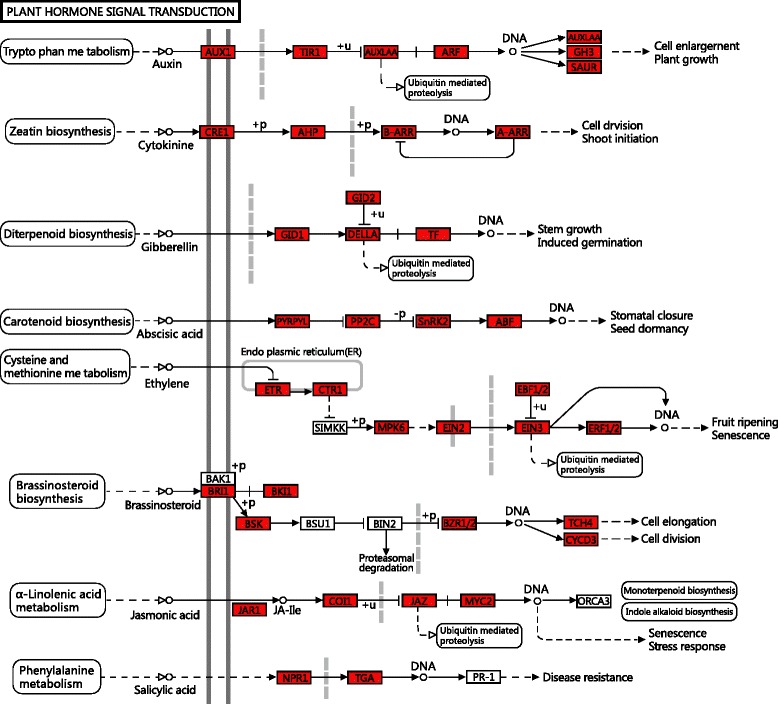
Unigenes involved in the plant hormone signal transduction pathway in SAM of Oriental hybrid lily. The genes in red were found by our Illumina sequencing

Based on a comparison against the KEGG database using BLASTx with an Evalue cutoff of < 10^−5^, of the 66,327 unigenes, 16,601 (25.03 %) had significant matches in the database and were assigned to 268 KEGG pathways (Table 4). The most representative pathways were ‘pyrimidine metabolism’ (278 members), ‘ribosome’ (289 members), ‘microbial metabolism in diverse environments’ (413 members), ‘biosynthesis of secondary metabolites’ (965 members), and ‘metabolism pathways’ (2067 members) (Table 4). These annotations provided an efficient resource for investigating pathways, functions, and processes involved in lily vernalization. In addition, we found unigenes involved in plant hormone signal transduction pathway, indicating that ABA, IAA, ethylene and GAs hormones may play important role in hormone signal transduction pathways of lily vernalization (Fig. 4).

**Table 4:** The KEGG biochemical pathways categorization of Oriental hybrid lily unigenes pathways

KEGG Categories	Mapped–KO	Unigene–NUM	Ratio of No.	Pathway–ID
DNA replication	32	42	0.25	ko03030
Oocyte meiosis	32	93	0.56	ko04114
N–Glycan biosynthesisn	32	54	0.33	ko00510
Proteasome	33	129	0.78	ko03050
Glycerophospholipid metabolism	34	73	0.44	ko00564
Arginine and proline metabolism	34	69	0.42	ko00330
Nucleotide excision repair	35	122	0.73	ko03420
Starch and sucrose metabolism	36	52	0.31	ko00500
Peroxisome	37	75	0.45	ko04146
Amino sugar and nucleotide sugar metabolism	37	74	0.45	ko00520
Viral carcinogenesis	39	81	0.49	ko05203
Plant hormone signal transduction	39	70	0.42	ko04075
Endocytosis	39	191	1.2	ko04144
Meiosis–yeast	41	89	0.54	ko04113
Photosynthesis	42	65	0.39	ko00195
HTLV–I infection	46	147	0.89	ko05166
mRNA surveillance pathway	47	140	0.84	ko03015
RNA degradation	49	139	0.84	ko03018
Cell cycle–yeast	53	103	0.62	ko04111
Ribosome biogenesis in eukaryotes	56	256	1.54	ko03008
Cell cycle	56	160	0.96	ko04110
Epstein–Barr virus infection	58	177	1.07	ko05169
Ubiquitin mediated proteolysis	60	149	0.90	ko04120
Pyrimidine metabolism	72	278	1.67	ko00240
Protein processing in endoplasmic reticulum	74	228	1.37	ko04141
Oxidative phosphorylation	81	192	1.16	ko00190
Purine metabolism	83	183	1.10	ko00230
RNA transport	89	184	1.11	ko03013
Biosynthesis of amino acids	96	250	1.51	ko01230
Spliceosome	99	230	1.39	ko03040
Ribosome	119	289	1.74	ko03010
Microbial metabolism in diverse environments	125	413	2.49	ko01120
Biosynthesis of secondary metabolites	333	965	5.81	ko01110
Metabolic pathways	781	1880	11.32	ko01100
Others	2243	8274	49.8

### Gene expression changes among different vernalization stages

**Fig. 5:**
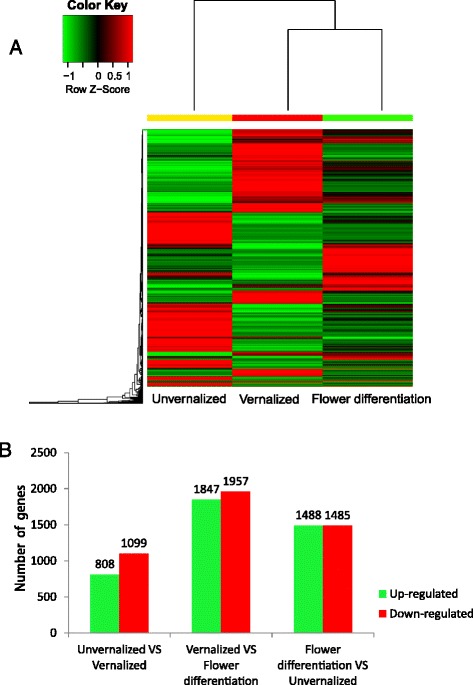
The expression of the gene changes among the different vernalization stages. **a**. The heat-map of the total differentially expressed genes (DEGs). Columns and rows in the heat maps represent samples and genes, respectively. Sample names are displayed below the heat maps. unvernalized, vernalized and flower differentiation results of treatments. Color scale indicates fold changes of gene expression. A fold change of ≥1 is shown in green (increased transcript abundance), a fold change of ≤ −1 is shown in red (decreased transcript abundance), and no change is indicated in black. The results show that 195 transcripts were differentially expressed between the unvernalized, vernalized and flower differentiation. **b**. Changes in gene expression profile among the different venalization stages. The number of up-regulated and down-regulated genes between unvernalized-vs-vernalized, vernalized-vs-flower differentiation and flower differentiation-vs-unvernalized are summarized. Between the unvernalized and vernalized Oriental hybrid lily libraries, there are 808 genes upregulated and 1099 genes down-regulated, while there are 1847 up-regulated genes and 1957 down-regulated genes between the vernalized and flower differentiation Oriental hybrid lily libraries, and 1488 upregulated genes 1485 down-regulated genes between the flower differentiation and unvernalized Oriental hybrid lily libraries

A RNA-Seq experiment was conducted using samples of unvernalized, vernalized and flower bud differentiation of Oriental hybrid lily and mapped the resulting reads to our reference transcriptome to determine which of the 66,327 genes were differentially expressed among the three stages, we filtered with an FDR ≤0.001 and |log_2_ (ratio)| ≥2; During the three stages, 195 DEGs was changed significantly. On one side, some genes were down-regulated from the unvernalized to vernalized stages, but up-regulated obviously at the flower bud differentiation. On the other side, it was showed that some genes increased expression at vernalized stage, but decreased transcript abundance at the flower diffferentiation stage. With an algorithm developed from the heat-map, the differentially expressed tags among the three samples were identified. Some genes were immediately expressed at the initial stage of unvernalized, while others were up-regulated subsequently indicating that transcription factors induced the expression of vernalization-related genes during the regulation of temperature signaling (Fig. 5a).

In addition, the vernalized and unvernalized libraries were compared and 1907 variable genes were found a total of 808 up-regulated and 1099 down-regulated genes were detected between the two libraries. There were also 1846 up-regulated and 1954 down-regulated genes between the vernalized and flower bud differentiation libraries, 1946 up-regulated and 1027 down-regulated genes between the unvernalized and flower bud differentiation libraries (Fig. 5b). This suggests that the differentiation of expressed genes between vernalized and flower bud differentiation is larger than that between the unvernalized and flower bud differentiation libraries, while the difference between and the unvernalized and vernalized libraries is the smallest of the three. That means, in Oriental hybrid lily, transcript abundance changed dramatically among the vernalization stages of vernalized to flower bud differentiation stage which the temperature response genes could be induced and expressed largely, and we should not ignore the expression of genes during the short-term of unvernalized to vernalized stage, because in this period, many important vernalization response genes were up- and down-regulated, they would earliest determine the plant to response to the low temperatureand to play instantaneous reflection.

**Fig. 6:**
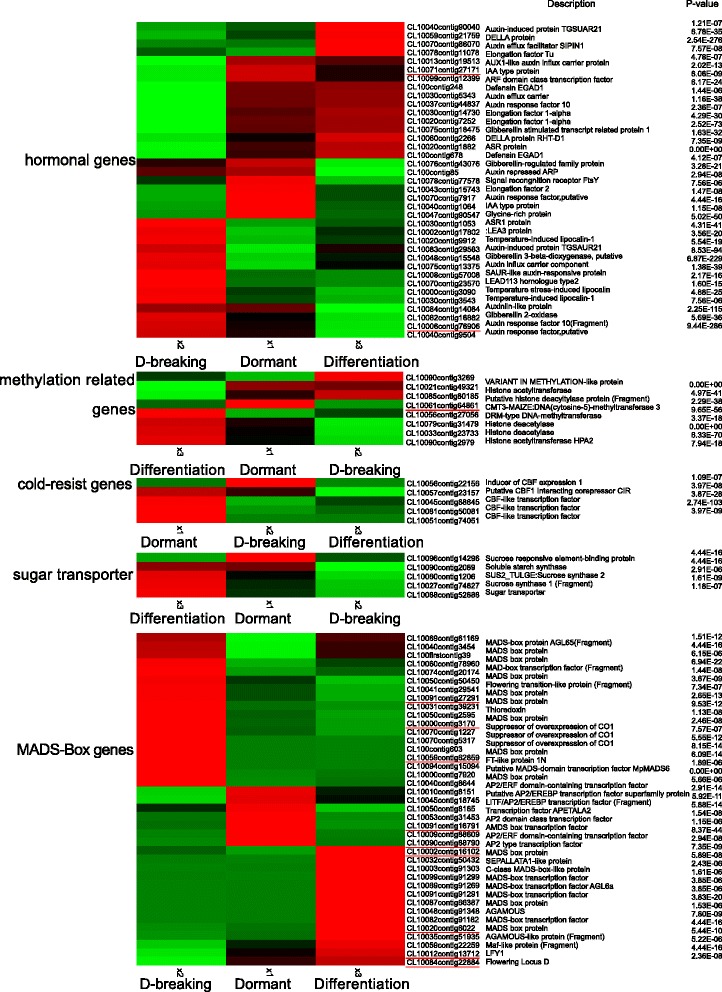
Heat-map of 93 differentially expressed genes involved in hormonal genes, methylation related genes and MADS-box genes of Oriental hybrid lily. They were differentially expressed between the unvernalized, vernalized and flower differentiation stages. The twelve genes highlighted in red underline were genes by qRT-PCR in Fig. 1

At the vernalized stage, genes whose transcript abundance exhibited highly dynamic changes (|log2 (ratio)| ≥ 4, Fig. 6) included MADS-Box genes (*FLC*, *SOC1*, *LFY* et al.), hormonal genes (*AUX*, *IAA1*, *ASR*, *ARF* et al.) and methylation related genes. Therefore, the change in expression patterns of distinct transcripts suggested the requirement of different development events from unvernalized to vernalized growth.

### qRT-PCR verifing gene expression profiles

To verify the genes expression in our Illumina sequencing analyses, 12 DEGs were selected for qRT-PCR using samples of unvernalized, vernalized and flower bud differentiation stages originally used for RNA-Seq, all of which are known to be related to low temperature, including the genes encoding *LoFLC* (Flowering locus C, Contig6022), *LoVRN2* (Vernalization 2, Contig27291), *LoFT* (FT-like protein, Contig62859), *LoAP2* (AP2-type transcription factor, Contig88790), *LoSOC1* (Suppressor of overexpression of CO1, Contig3170), *LoLFY* (LEAFY, Contig13712), *LoVRN1* (*Vernalization 1*, Contig16102), *LoSVP* (Shot vegetative phase, Contig16791), *LoFLD* (Flowering locus D, Contig22884), *LoCMT* (CMT-type DNA methyltransferase, Contig64861), *LoARF10* (Auxin response factor 10, Contig76906), *LoIAA* (IAA type protein, Contig27171). The Ct values of the *LoTIP* in all samples ranged from 24.0 to 26.0. All 12 transcripts showed the same expression pattern as the *in silico* differential analysis results from high-throughput sequencing.

These genes were selected for their key roles in regulating low temperature signal transcription, vernalization responses, and cold acclimation. The results presented in Fig. 1 showed that the expression levels of flowering integrating factors (*LoFT*, *LoLFY*, *LoSOC1*) were higher in vernalized and flower differentiation samples than in unvernalized sample, which indicated that the three genes may play key roles in floral transition. The expression of vernalization-related genes (*LoVRN2*) decreased sharply in vernalized sample than in unvernalized sample, which indicated that *LoVRN2* may play an important role in floral induction. The expression of *LoFLC* was higher in 42-vernalized stage when the *LoSOC1* gene highly expressed than in 35-vernalized stage, which indicated that *LoFLC* may delay the floral transition. Growth hormone-related genes (*LoAP2*, *LoIAA1*, *LoARF10*) were higher in unvernalized sample than in vernalized and flower differentiation samples and DNA methylation related genes (*LoCMT*, *LoFLD*) expressed differently in all stages. We predicted that the bulb has played an important role in for the Oriental hybrid lily adaptation to low temperature and completing vernalization.

**Fig. 7:**
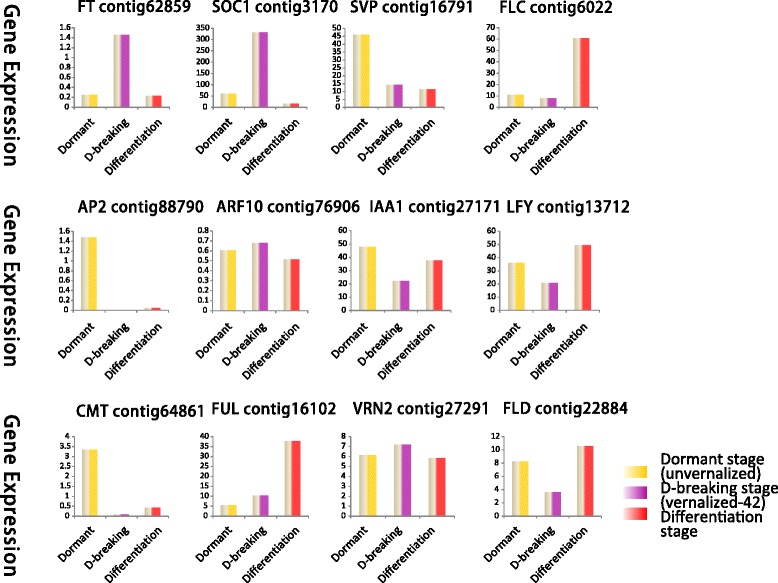
The expression profiles of 12 transcripts in Oriental hybrid lily by RNA-seq. The dormant stage, dormancy-breaking stage, differentiation stage correspond to unvernalized period, vernalized-42-days period and differentiation period in Fig. 1, respectively. And every two corresponding periods in Fig. 1 and Fig. 7 are in the same color

Another new figure (Fig. 7) of the expression profiles of 12 transcripts in Oriental hybrid lily by RNA-seq was made in order to correspond to the expression of the same genes by qRT-PCR in Fig. 1. The symbol’V-42′ in Fig. 1 is on behalf of the samples that were obtained after 42-days vernalization, which happens to be the timing of bulbs’dormancy-breaking stage in Fig. 7 It is found that the expression of *LoSVP*, *LoFLC*, *LoVRN2* genes by qRT-PCR are not agreed with those from RNA-seq. The expression of *LoSVP* gene in Fig. 7 was highest in the dormant stage, however the gene expressed highest in the V-42 period in Fig. 1. The expression of *LoFLC* gene increased in the differentiation stage in Fig. 7 but decreased in differentiation stage in Fig. 1. The *LoVRN2* gene was in lower expression level in the dormancy-breaking stage and differentiation stage in Fig. 1, but it kept expressing highly in Fig. 7.

### Phylogenetic analysis of DNA sequence and deduced amino acid sequence of LoSVP and LoVRN1

In our EST database, the highest expressing transcript was homologous to shot vegetative phase gene (*LoSVP*) and vernalization 1 gene (*LoVRN1*). *SVP* encodes a nuclear protein that acts as a floral repressor and represses *FT* expression via direct binding to the vCArG III motif in the *FT* promoter. For further analysis, we thus screened the complete sequence of *LoSVP* and *LoVRN1* from the Oriental hybrid lily cDNA library by RACE. The putative transcription start and stop sites of *LoSVP* and *LoVRN1* were amplified with gene-specific primers designed from the partial cDNA sequence.

**Fig. 8:**
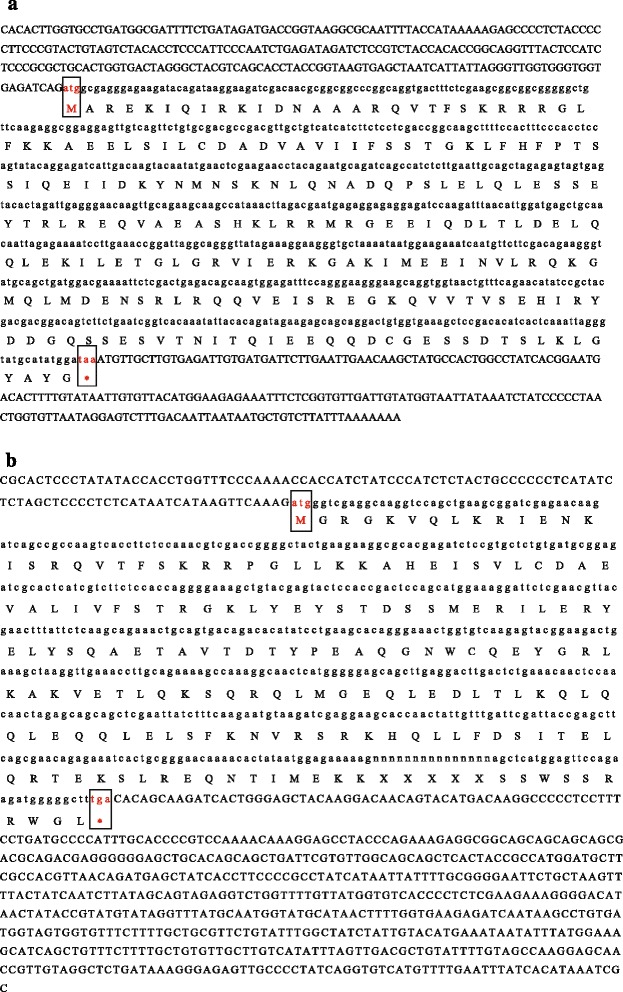
Analysis of cDNA sequence encoding a *SVP* protein **a** and a *VRN1* protein **b** isolated from Oriental hybrid lily. Nucleotide and deduced amino acid sequences of the *SVP* and *VRN1* protein

The predicted molecular mass and pI value of *LoSVP* were 36.7 kDa and 4.85, respectively. The transcripts defined the *LoSVP* full-length cDNA sequence with putative open reading frames of 675 bp encoding predicted proteins of 224 amino acids (Fig. 8a). The DNA sequence and deduced amino acid sequence of Oriental hybrid lily *LoSVP* showed similarities to members of *SVP* family from *Arabidopsis*, *Elaeis* and other plant species. Oriental hybrid lily *LoSVP* contains the highly conserved MADS-box from amino acids 3–57 and K-box from amino acids 86–176 conserved domains as detected by the Motif scan program implemented in the PROSITE database [37] and belonged to TypeII MADS-box protein. No signal peptide was predicted by SignalP (http://www.cbs.dtu.dk/services/SignalP-2.0/), which is consistent with *SVP* found in other species. SVP was located in Nuclear by analyzing for subcellular localization (http://wolfpsort.seq.cbrc.jp/).

The predicted molecular mass and pI value of *LoVRN1* were 32.5 kDa and 6.54, respectively. The transcripts defined the *LoVRN1* full-length cDNA sequence with putative open reading frames of 561 bp encoding predicted proteins of 186 amino acids (Fig. 8b). The DNA sequence and deduced amino acid sequence of Oriental hybrid lily *LoVRN1* showed similarities to members of *VRN1* family from *Lilium longiflorum*, *Tulipa gesneriana* and other plant species. Oriental hybrid lily *LoVRN1* contains the highly conserved MADS-box from amino acids 3–57, K-box from amino acids 88–185 conserved domains and EF-hand calcium-binding domain from amino acids 60–72 conserved domains as detected by the Motif scan program implemented in the PROSITE database [16] and belonged to TypeIIMADS-box protein. A signal peptide between 43 to 44 amino acids was predicted by SignalP, which is consistent with signal peptides found in *VRN1s* from other species. *VRN1* was located in Nuclear by analyzing for subcellular localization.

**Fig. 9:**
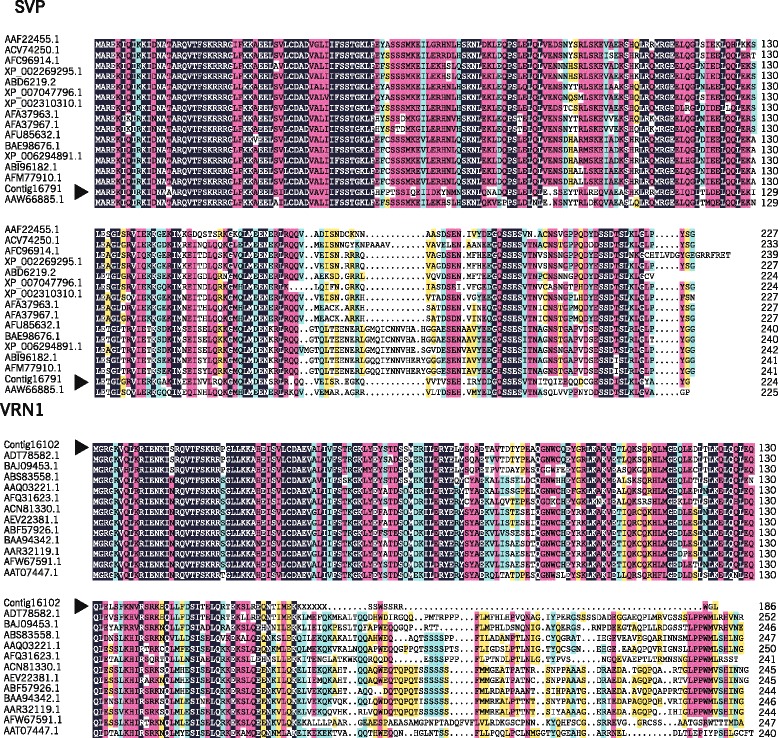
Protein sgquence multiple alignment of the deduced amino acid sequences of *LoSVP* and *LoVRN1* with other plant *SVPs* and *VRN1s*. The GenBank accession numbers of *SVP* are as follows: *Theobroma cacao* (XP 007047796), *Populus trichocarpa* (XP 002310310), *Paulownia kawakamii* (AAF22455.1), *Vitis vinifera* (XP 002269295), *Vitis vinifera* (AFC96914.1), *Malus domestica* (ABD66219.1), *Brassica rapa* (ABI96182.1), *Brassica napus* (AFM77910.1), *Capsella rubella* (XP 006294891), *Arabidopsis thaliana* (AFU85632.1), *Arabidopsis thaliana* (BAE98676.1), *Petunia* × *hybrida* (ACV74250.1), *Actinidia chinensis* (AFA37963.1), *Actinidia chinensis* (AFA37967.1), *Elaeis guineensis* (AAW66885.1). The GenBank accession numbers of *VRN1* are as follows: *Festuca arundinacea* (ACN81330.1), *Lolium perenne* (AEV22381.1), *Triticum aestivum* (ABF57926.1), *Dendrocalamus latiflorus* (AAR32119.1), *Oryza saliva* (Baa94342.1), Zea mays (AFW67591.1), Elaeis guineensis (AAQ03221.1), *Alpinia oblongifolia* (ABS83558.1), *Cymbidium ensifolium* (AFQ31623.1), *Vitis vinifera* (AAT07447.1), *Tulipa gesneriana* (BAJ09453.1), *Lilium longiflorum* (ADT78582.1). *LoSVP* and *LoVRN1* are indicated with an arrow at the left

**Fig. 10:**
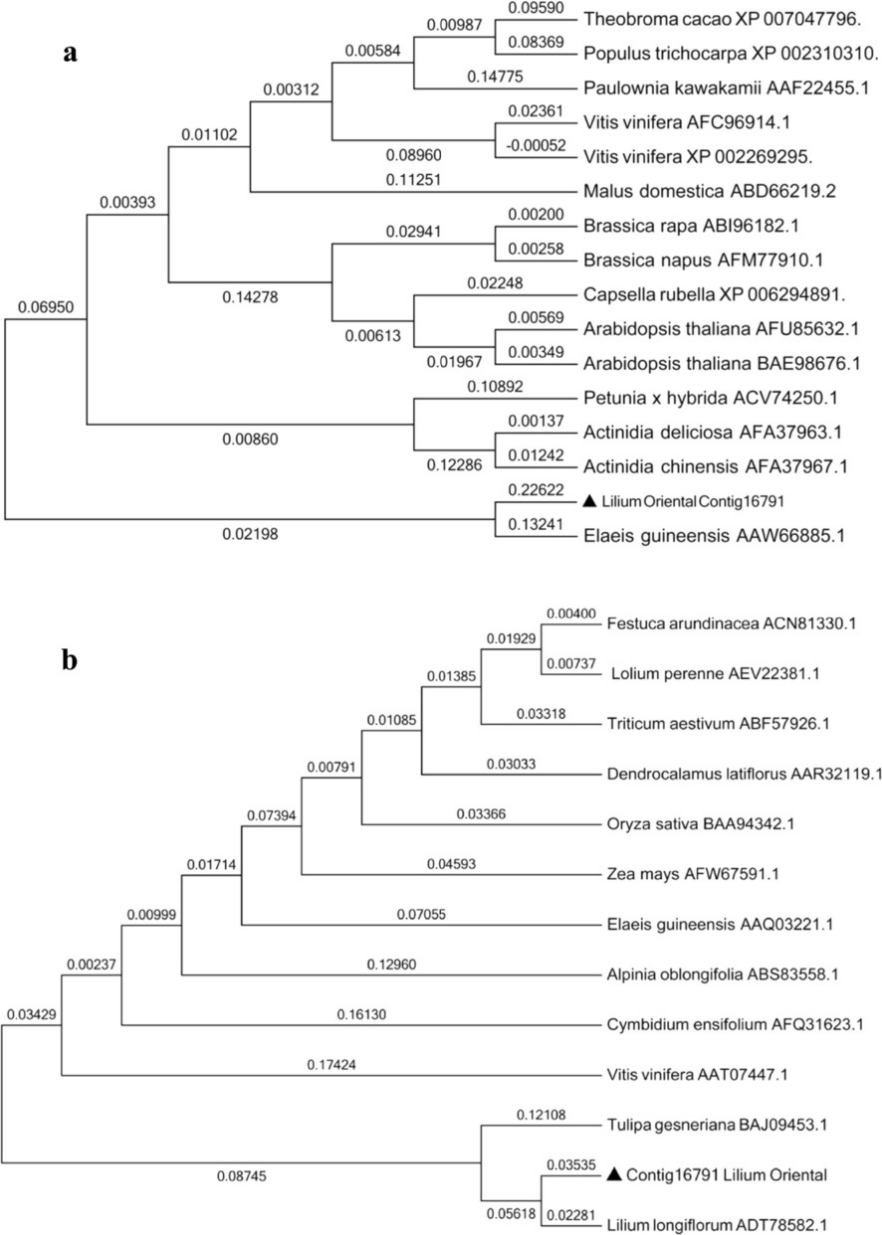
Phylogenetic tree of L. Oriental *LoSVP* with 15 other plant SVPs and *LoVRN1* with 12 other plant *VRN1s* based on deduced amino acid sequences

An alignment of the deduced *LoSVP* and *LoVRN1* with other plants *SVPs* and *VRN1s* showed that *LoSVP* and *LoVRN1* shared high sequence identities with *Elaeis guineensis LoSVP* and *Tulipa gesneriana LoVRN1*, respectively (Fig. 9). Furthermore, an amino acid neighbor-joining tree was constructed for *LoSVP* (Fig. 10a). Fifteen amino acid sequences of various plants were acquired from the Genbank database. A pairwise comparison of the degree of amino acid identity was performed for the *SVPs*. The identity shows high conservation of *SVPs* in plants. In the *SVP* amino acid tree, the *SVP* of L. Oriental (*LoSVP*) is closely related to *Elaeis guineensis*, with high bootstrap value. The *SVP* of lily clustering with other monocots is a sister to a clade of other dicots. The phylogeny of SVP group indicateds that a single-class *SVP* gene existed before the divergence of dicots and monocots [16]. Twelve amino acid sequences of various plants were acquired from the Genbank database to construct an amino acid neighbor-joining tree for *LoVRN1* (Fig. 10b). A pairwise comparison of the degree of amino acid identity was performed for the *VRN1s*. The identity shows high conservation of *VRN1s* in plants. In the *VRN1* amino acid tree, the *VRN1* of L. Oriental (*LoVRN1*) is closely related to *Lilium longiflorum*, *Tulipa gesneriana*, with high bootstrap value.

### Ectopic expression of *LoVRN1* causes early flowering and homeotic conversion of flowers in transgenic Arabidopsis plants

**Fig. 11:**
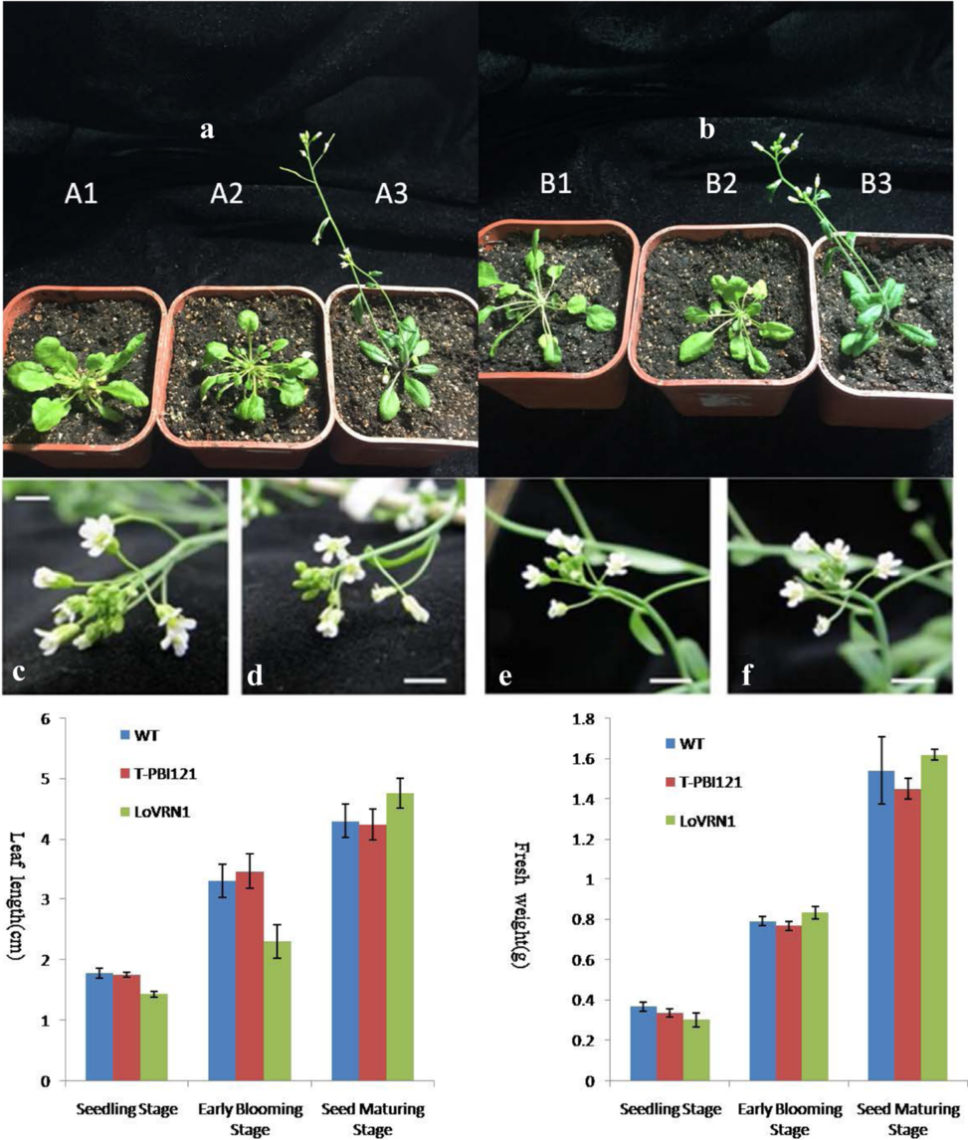
Phenotypic analysis of Arabidopsis mutants ectopically expressing LoVRN1. **a**/**b** The 34-day-old 35S:: *LoVRN1* plants (right—A3, B3) grown on soil flowered significantly earlier than wild plants (left—A1, B1) after producing only six curled rosette leaves, and flowered around 20d after germination. Flowers with 5 petals **d**, 6 petals **e**, **f**), and 7 petals **c** are shown. Scale bar is 3 mm

To investigate the function of *LoVRN1*, the cDNAs of *LoVRN1* gene driven by the cauliflower mosaic virus (CaMV) 35S promoter were transformed into Arabidopsis plants for functional analysis. Ten independent 35S:: LoVRN1 transgenic Arabidopsis T1 plants which flowered around 20d after germination were obtained, and showed identical phenotypes by flowering earlier than wild-type plants and great differences in the flower bud differentiation and flower organ development process. There mainly exhibited 5, 6 or 7 valve performance traits in the late growth process of overexpressing *LoVRN1* transgenic plants petals, while the wild-type plants were 4 petals (Fig. 11). In addition, by blade length and plant fresh weight statistical analysis, it showed some differences between overexpression *LoVRN1* transgenic plants and wild-type plants (Fig. 11). The blade length of transgenic Arabidopsis plants was less than that of wild-type plants before the seed maturing stage. The plant fresh weight of transgenic Arabidopsis plants increases sharply than that of wild-type plants since the early blooming stage.

### Ectopic expression of *LoSVP* causes late flowering in transgenic Arabidopsis plants

**Fig. 12:**
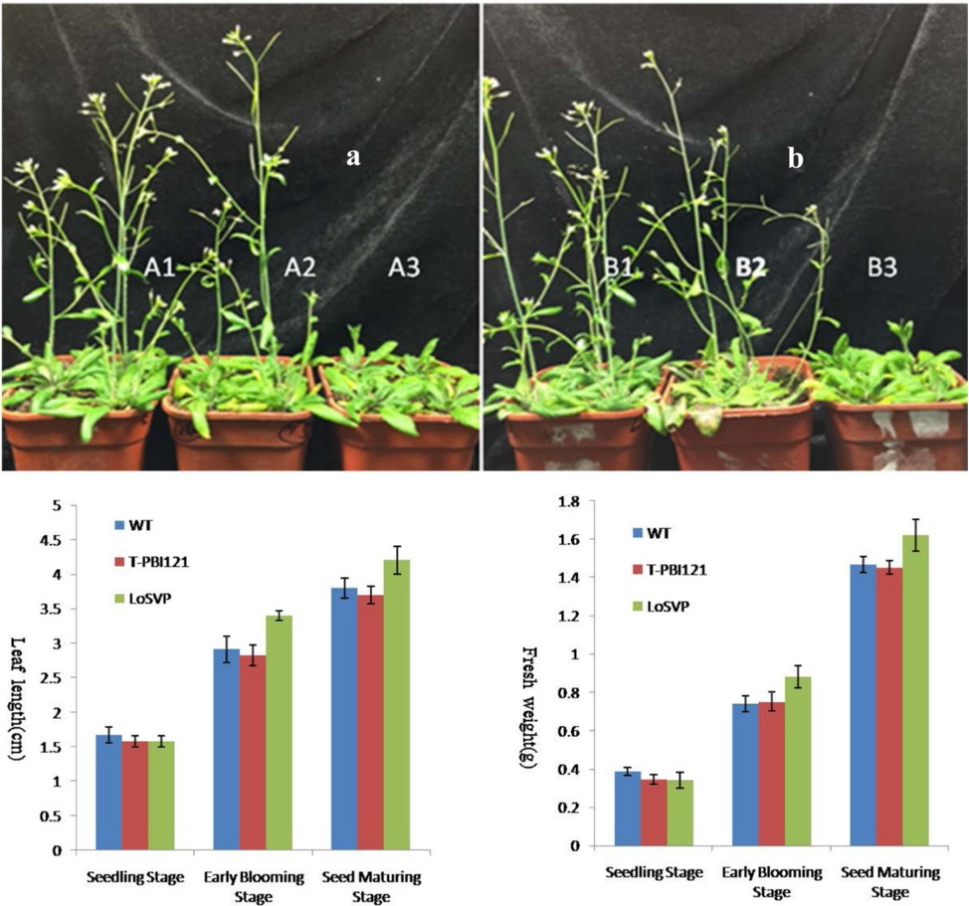
Phenotypic analysis of Arabidopsis mutants ectopically expressing LoSVP. **a**/**b**) The 34-day-old 35S:: *LoSVP* plants (right—A3, B3) grown on soil with producing only six curled rosette leaves flowered significantly later than wild plants (left—A1, B1) and the transgenic pbi121 without any genes plants (left—A2,B2)

To investigate the function of *LoSVP*, the cDNAs of *LoSVP* gene driven by the cauliflower mosaic virus (CaMV) 35S promoter were transformed into Arabidopsis plants for functional analysis. Eight independent 35S:: LoSVP transgenic Arabidopsis T1 plants were obtained and showed identical phenotypes by flowering later than wild-type plants and some differences in blade length and plant fresh weight statistical analysis (Fig. 12). The blade length of transgenic Arabidopsis plants was always less than that of wild-type plants until the seed maturing stage. In Table. 5, the mean value of the leaf length of the *LoSVP* transgenetic plants is 1.58 cm, which is less than that of wild-type plants of 1.67 cm in the seedling stage. The mean value of the fresh weight of the LoSVP transgenetic plants is 0.34 g, which is also less than that of wild-type plants of 0.39 g in the seedling stage. In the same way, we can notice that the blade length and fresh weight data of transgenic Arabidopsis plants were more than those of wild-plants both in the early blooming stage and seed maturing stage, which were marked in red in Table 5. Similarly, the same conclusion can be obtained in Fig. 12.

**Table 5:** Effect of different transgenic treatment on morphological changes during Arabidopsis plants development

Morphological traits	Treatment	Seedling Stage	Early Blooming Stage	Seed Maturing Stage
Leaf length (cm)	WT	1.78 ± 0.08^b^	3.3 ± 0.27^d^	4.30 ± 0.27^e^
	T–PBI121	1.75 ± 0.04^b^	3.46 ± 0.29^d^	4.24 ± 0.25^e^
	LoVRN1	1.43 ± 0.04^a^	2.30 ± 0.27^c^	4.76 ± 0.25^f^
Fresh weight (g)	WT	0.37 ± 0.02^a^	0.79 ± 0.02^b^	1.54 ± 0.17^e^
	T–PBI121	0.34 ± 0.02^a^	0.77 ± 0.02^b^	1.45 ± 0.05^c^
	LoVRN1	0.30 ± 0.04^a^	0.83 ± 0.03^b^	1.62 ± 0.03^e^
Leaf length (cm)	WT	1.67 ± 0.12^a^	2.91 ± 0.19^b^	3.80 ± 0.14^d^
	T–PBI121	1.58 ± 0.08^a^	2.82 ± 0.15^b^	3.70 ± 0.12^d^
	LoSVP	1.58 ± 0.08^a^	3.40 ± 0.07^c^	4.20 ± 0.2^e^
Fresh weight (g)	WT	0.39 ± 0.02^a^	0.74 ± 0.04^b^	1.47 ± 0.04^d^
	T–PBI121	0.34 ± 0.03^a^	0.75 ± 0.05^b^	1.45 ± 0.04^d^
	LoSVP	0.34 ± 0.04^a^	0.88 ± 0.06^c^	1.62 ± 0.08^e^

Note: ^Y^ Date are means of eight independent biological replicates (±SEM); ^X^ Mean separation within columns by Duncan’s multiple range test at *P* ≤ 0.05 (lowercase letter)

## Discussion

### Plant hormone signal transduction in vernalization

Plant hormones could affect diverse developmental processes and were small organic molecules [38]. In our study, we found that plant hormones were involved in the flower differentiation of lily as ARF10, IAA1 genes and ethylene gene *AP2* expressed differently (Fig. 1) among the three samples, especially at the dormancy-breaking bulbs stored at 4 °C indicates that auxin is produced in the shoot during the cold treatment period, which leads to shoot emergence, floral stalk elongation and finally the flowering. These data will be valuable resources for investigations towards understanding specific auxin responses or a subset of auxin responses as well as hormonal crosstalk during the floral induction phase in vernalization. Previous study showed that IAA was present in the shoot apexes throughout the floral induction process, gradually concentrating in the shoot apical meristem (SAM). That study also suggested that IAA was the significant agent for floral induction, and that SAM might be the place of the main action [39]. Finally, an appropriate amount of IAA in the SAM and normal polar auxin transport are essential for floral induction and differentiation in Lily. In our study, we found growth hormone-related genes (*LoAP2*, *LoIAA1*, *LoARF10*) were higher in unvernalized sample than in vernalized and flower differentiation samples. Another study also showed that both IAA and ethylene application inhibit flower induction in the short-day plant Pharbitis nil and the inhibitory effect of IAA on flowering is not physiological but is connected with its effect on ethylene biosynthesis [40]. Other hormones, such as GAs and cytokinins might also be involved in flower development through changes in concentration or by interaction with other hormones during the period of storage at cold or during growth of shoots of bulbs in greenhouses [39]. Therefore, it may have important significance to find more genes in the GA floral induction pathway in later researches of Lily.

### Basal metabolism and DNA methylation play important roles in lily vernalization process

In early twentieth century, scientists proved that the nutritional status of plants could influence plant flowering through a lot of experiments. The results of the study showed that the gene expression in starch, carbohydrate metabolism and nitrogen metabolism, were significantly up- regulated during vernalization. *FLD* encoding a plant ortholog of the human protein Lys-Specific Demethylase1 (*LSD1*), is involved in H3K4 demethylation, [39], involed in positive regulation of flower development, inflorescence development, auxin biosynthetic process, cotyledon development, histone deacetylation, and oxidation-reduction process.. Lesions in *FLD* result in hyperacetylation of histones in *FLC* chromatin, up-regulation of *FLC* expression and extremely delayed flowering [40]. Although cross talk between demethylation and histone deacetylation has previously been indicated to modulate gene expression in mammalian cells, the direct association of *FLD* and *CMT* with a histone demethylase has not been reported. In our study, we found that the expression of DNA methylation genes *LoCMT* and *LoFLD*, which expressed differently among the three stages. These results suggest that *CMT* and *FLD* play an important role in the interplay between histone deacetylation and DNA methylation in transcriptional regulation. This is a solid foundation for further characterization of the *LoCMT* and *LoFLD*. The expression of vernalization-related genes (*LoVRN2*) decreased sharply in vernalized sample than in unvernalized sample, which indicated that *LoVRN2* may play an important role in floral induction. While previous studies in wheat and barley have revealed the functional role of histone modification in setting *VRN1* expression [43]. Here, we are interested in determining whether the cold-induced expression of the *LoVRN2* gene is associated with a change in DNA methylation, which will be my next research. Our study provides evidence of the role of DNA methylation in vernalization for Lily, which is necessary for the transition to reproductive growth.

### Genetic potential of LoVRN1 and LoSVP applied in lily

*LoVRN1* and *LoSVP* had important roles in the growth and development of plants, both could promote plant growth and transition from vegetative to reproductive growth, namely impacting flower transition [41, 42, 44]. Combined with previous transcriptome analysis, we speculated that *LoVRN1* and *LoSVP* played important roles in lily flowering regulation. To further explore the molecular mechanism of lily vernalization process and understand the relationship between vernalization and *LoVRN1* and *LoSVP*, and the relationship between *LoVRN1* and *LoSVP* and flowering time, we cloned *LoVRN1* and *LoSVP* genes. Through bioinformatics analysis and transgenic analysis, we found *LoVRN1* could response to lily vernalization process and promote early flowering. In addition, there was a certain role of *LoVRN1* gene in changing the type of lily. On the other side, *LoSVP* had a role in delaying flowering time. Due to the current genetic transformation based on genetic engineering and genetic modification techniques applied in a wide range of production and some crops, it was possible to overexpress *LoVRN1* and *LoSVP* in lily to change the flowering time of lily. Previous studies showed that the up-regulation of *VRN1* during winter is required to maintain low transcript levels of *VRN2*, accelerate the induction of *FT* in the leaves, and regulate a timely flowering in the spring. What just conforms this hypothesis in our study is that the expression of vernalization-related genes (*LoVRN2*) decreased sharply in vernalized sample than in unvernalized sample. But this hypothesis was reinforced by the observation that vernalization promotes an active chromatin state in *VRN1* regulatory regions but not in those of *VRN2* or *FT* [45]. Another study also demonstrates that the down-regulation of *VRN2* during vernalization does not require the presence of VRN1 [46]. Therefore, it will be an important research direction of how genes interact on each other during vernalizaiton. The understanding of functions of genes and the regulatory mechanisms involved in the initiation of flowering, especially the vernalization promotion pathway, will be beneficial to promote flowering of lily.

## Conclusion

In conclusion, this study provides the first set of comprehensive floral transcriptome data in Oriental hybrid lily ‘Sorbonne’. 66,327 Orential hybrid lily unigenes that provide information on gene expression patterns involved in the plant’s vernalization and flower development were generated. From a functional categorization analysis of unigenes, followed by realtime qRT-PCR analysis, we found a high expression of genes involved in the low temperature response and flower development, especially we found that plant hormones signal transduction, DNA methylation were involved in the process of lily responses to vernalization under low temperature. The cDNAs identified from the cDNA library include two vernalizaiton-related genes (*LoSVP* and *LoVRN1*), which appear to be promising candidates for playing key roles in the development and response of flowering in Oriental lily plants. One of the new practice of this manuscript was to collect a sample for transcriptome sequencing and comparison when the bulb’s apical meristem was in the time of floral transition in which the expression of floral transition key gene SOC1 increased sharply by the qRT-PCR (Fig. 1) and the apical meristem had not converted into the morphological differentiation process, which helped to obtain more genes playing key roles in the floral induction pathways, such as vernalization pathway, gibberellic acid pathway, autonomous pathway and so on. Another new practice of this manuscript was to detect the relative expression of 12 genes, all of which are known to be related to low temperature, by qRT-PCR using samples which be collected every other week in vernalization stage besides other two samples in unvernalized stage and flower bud differentiation stage. The upstream and downstream relationship between different genes were forecasted by the analysis of genes’ expression levels in a wide range of time (Fig. 4, Fig. 1). The above two points both promoted establishing and perfecting the molecular mechanisms of floral induction pathway by vernalization.

## Abbreviations

BLAST: Basic Local Alignment Search Tool
COG: Clusters of Orthologous Groups
DGE: Digital Gene Expression
EST: Expressed Sequence Tag
FDR: False Discovery Rate
GO: Gene Ontology
KEGG: Kyoto Encyclopedia of Genes and Genomes Pathway
NCBI: National Center for Biotechnology Information
qRT-PCR: Real-time Quantitative Reverse Transcription Polymerase Chain Reaction
RACE: Rapid Amplification of cDNA Ends
RPKM: Number of Reads per Kilobase per Million Clean Reads
SAM: Shoot Apical Meristem
